# “Back to the Future”: Time for a Renaissance of Public Health Engineering

**DOI:** 10.3390/ijerph16030387

**Published:** 2019-01-29

**Authors:** Richard J. Gelting, Steven C. Chapra, Paul E. Nevin, David E. Harvey, David M. Gute

**Affiliations:** 1 Division of Global Health Protection, Center for Global Health, Centers for Disease Control and Prevention, Atlanta, GA 30329 USA; rug7@cdc.gov; 2 Department of Civil and Environmental Engineering, Tufts University, Medford, MA 02155, USA; steven.chapra@tufts.edu; 3 Department of Global Health, University of Washington, Seattle, WA 98195, USA; penevin@uw.edu; 4 Division of Sanitation Facility Construction, Indian Health Service, Rockville, MD 20857, USA; david.harvey@ihs.gov

**Keywords:** engineering, public health, curriculum proposals

## Abstract

Public health has always been, and remains, an interdisciplinary field, and engineering was closely aligned with public health for many years. Indeed, the branch of engineering that has been known at various times as sanitary engineering, public health engineering, or environmental engineering was integral to the emergence of public health as a distinct discipline. However, in the United States (U.S.) during the 20th century, the academic preparation and practice of this branch of engineering became largely separated from public health. Various factors contributed to this separation, including an evolution in leadership roles within public health; increasing specialization within public health; and the emerging environmental movement, which led to the creation of the U.S. Environmental Protection Agency (EPA), with its emphasis on the natural environment. In this paper, we consider these factors in turn. We also present a case study example of public health engineering in current practice in the U.S. that has had large-scale positive health impacts through improving water and sanitation services in Native American and Alaska Native communities. We also consider briefly how to educate engineers to work in public health in the modern world, and the benefits and challenges associated with that process. We close by discussing the global implications of public health engineering and the need to re-integrate engineering into public health practice and strengthen the connection between the two fields.

## 1. Background

Public health and engineering were closely aligned as professional fields for many years; indeed, engineering was integral in the emergence of public health as a distinct discipline from clinical medicine [1]. However, the practice and profession of environmental engineering have become partially separated from public health in the latter half of the 20th century in the United States (U.S.) [2]. Several factors contributed to this separation, including an evolution in leadership roles within public health; increasing specialization within public health, including the formation of the sanitarian/environmental health field, to which some tasks migrated that were formerly within public health engineering; and the emerging environmental movement, which led to the creation of the U.S. Environmental Protection Agency, with its emphasis on human health and the natural environment. In this paper, we consider these factors in turn, and also highlight an example of public health engineering in current practice in the U.S. We also consider how to educate public health engineers in the modern world, and the global implications of public health engineering.

Engineering is obviously found in other content areas and occupations within public health beyond environmental applications. For example, biomedical engineering is prominent within the Food and Drug Administration (FDA), as is safety engineering and industrial hygiene in the National Institute of Occupational Safety and Health (NIOSH) [3]. In this paper, however, we specifically focus on the aspects of engineering that were most closely aligned with the emergence of public health, and that have been known at various times as sanitary engineering, public health engineering, and environmental engineering. We believe that there is a current need to strengthen the connection between this type of engineering and the practice of public health, and an appropriate moniker for this profession is “public health engineering”.

## 2. The Divergence of Public Health and Engineering in the Latter Half of the 20th Century

**Figure 1:**
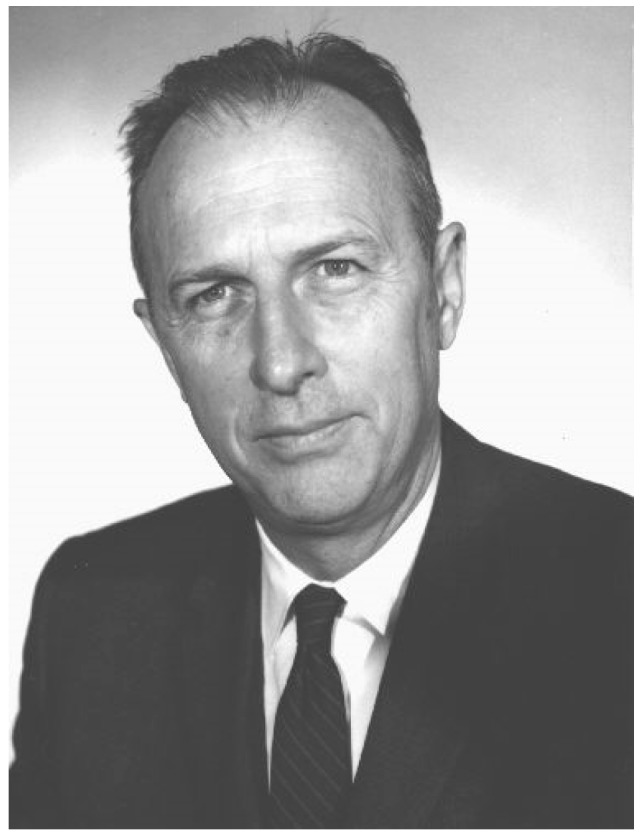
Mark Hollis (U.S. Centers for Disease Control, CDC, Public Health Image Library ID# 1304).

Public health has always been, and remains, an interdisciplinary field, but leadership within the field has evolved over time. Starting in the late 19th century, with the emergence of bacteriology and laboratory analysis, a medical perspective became increasingly prominent in public health [4]. This shift in leadership is evident in the U.S., and the evolution of roles is apparent in the history of the U.S. Centers for Disease Control and Prevention (CDC), although this transition within public health clearly started earlier. The first director of the CDC when it initially became a permanent U.S. government agency (the Communicable Disease Center) in 1946, Mark Hollis, ScD (Figure 1), was an engineer [3], a fact little known today, even amongst CDC staff. The agency, which grew out of the Malaria Control in War Areas program created during World War II, was largely staffed by engineers and entomologists during its initial years, reflecting the professions working in the control of mosquito-borne diseases [5]. A review of the history of CDC notes that there were only seven physicians on the staff out of 369 employees when the agency was formed in 1946 [6]. At the time, public health was not always considered the most preferable career option for physicians in the U.S. [7].

As many mosquito-borne diseases were largely controlled in the U.S.—the nation was considered malaria free by 1951 [8]—CDC leaders recognized that the agency’s focus needed to expand to include all communicable diseases, and that “To survive, it had to become a center for epidemiology” [5]. This expansion led to the development and refinement of public health surveillance programs [9]. The Epidemic Intelligence Service (EIS) program was started within CDC in 1951, with the stated intent to train epidemiologists to counter the possibility of biological warfare during the Korean War [5]. The program also fulfilled a vision within CDC to create a cadre of public health epidemiologists to investigate disease outbreaks of any kind. The first EIS class of 23 included 22 physicians and a single sanitary engineer [10]. Physicians continue to be prominent within the program, and made up 78% of the nearly 2500 graduates during the first 50 years of the EIS from 1951–2000, while only a handful of engineers participated during this period [10]. The EIS program helped to change negative perceptions among physicians about public health careers, and EIS graduates went on to become leaders and administrators in public health programs, in both the CDC and at the state level. Indeed, since 1953, all CDC directors have been physicians.

At about the same time that physicians were becomingly increasingly prominent in public health in the U.S., further differentiation of other public health professions was also taking place. The term “Sanitarian” was used in the early part of the 20th century as a general descriptor for all public health professionals, including sanitary engineers and physicians working in public health [11,12,13,14]. Later, it was sometimes used to describe those working in the field of public health engineering, but without specific engineering education or experience [15]. By the middle of the 20th century, use of the designation had evolved to describe those engaged in more specific environmental health tasks, especially related to ensuring safe food, water, and sanitation [16]. In 1961, the American Public Health Association (APHA) proposed a model act for registering Sanitarians, who were defined as “(persons) who by education and experience in the physical, biological, and sanitary sciences, (are) qualified to carry out educational, investigational and technical duties in the field of sanitation.” These tasks were previously considered to be part of the realm of the public health engineer, but were now migrating to the emerging profession of environmental health. More recently, the terms Environmental Health Professional, Environmental Health Scientist or Environmental Health Officer have largely replaced the designation of Sanitarian. Nonetheless, the practice of environmental health has always been closely linked to public health (indeed, the proceedings of some early APHA meetings were published in a journal entitled “The Sanitarian”), whereas the practice of public health engineering has largely become divorced from public health.

While these developments were taking place within the field of public health, another outside force that would also shape the relationship between engineering and public health soon emerged. The publication of the book “Silent Spring” by Rachel Carson in 1962 catalyzed the environmental movement, and raised awareness among both the public and policy makers of the impacts of human action on the environment. The environmental movement incorporated not only the concept of preservation of the natural world, but also enforcement to punish those who pollute it [17]. These attitudes eventually led to the creation of the U.S. Environmental Protection Agency (EPA) in 1970, which consolidated numerous federal programs dealing with environmental pollution issues under one organization. Consequently, most of the engineers working within the U.S. Public Health Service (USPHS) at the time moved to the EPA. Many states mirrored this federal action, moving environmental activities out of health departments [18]. As a result, engineers and engineering as a profession increasingly became aligned with environmental rather than public health issues. This transition also reinforced the emerging use of the term environmental engineer to describe practitioners in the field, replacing the earlier descriptors sanitary engineer and public health engineer.

Although public health engineering may have largely lost recognition as a profession, the practice of it is alive and well in some programs in the U.S. We offer the following case which explores the contributions of the intellectual drivers of public health engineering as it shaped the activities of the USPHS and the Indian Health Service.

## 3. A Case Study of Public Health Engineering: The Indian Health Service Experience in Native American and Alaska Native Communities

One excellent, but not widely known, example of public health engineering in practice in the U.S. is the Sanitation Facilities Construction (SFC) Program within the Indian Health Service (IHS), and related tribal programs. IHS is an Operating Division within the U.S. Department of Health and Human Services (DHHS), and is responsible for providing federal health services, including public health services, for federally recognized American Indian and Alaska Native (AI/AN) communities.

The Indian Sanitation Facilities Act (Public Law (PL) 86–121, commonly referred to as PL 86–121) was signed into law in 1959 in recognition of the lack of water and sanitation services for tribal communities. Through this law, the U.S. Congress authorized the Surgeon General to construct essential sanitation facilities for Indian homes, communities and lands. This authorization was to help ensure Indian homes and communities would have access to safe drinking water supply and sewage disposal systems which in turn would positively impact health. As a result of this authorization the Division of Sanitation Facilities Construction (DSFC) within the IHS was created. PL 86–121 also directed the Surgeon General of the USPHS to consult with and encourage the participation of tribes impacted in developing sanitation facility projects [19]. This was one of the first federal requirements for tribal participation in Native American programs, and DSFC has worked with tribes since the beginning of the program to identify needs and projects and ensure support for operation and maintenance. Under the 1993 Indian Self-Determination and Education Assistance Act (Public Law 93–638, commonly referred to simply as “638”), 20 tribal governments have taken on some DSFC program functions as of 2015. These functions are still at least partially funded by IHS, and still maintain the principles of a public health based engineering program.

The work of the DSFC has made significant progress since its creation in 1959, increasing the proportion of AI/AN (American Indian/Alaska Native) homes without essential water and sanitation facilities from less than 20% in 1955 to over 90% in 2012 [20]. Nonetheless, significant challenges remain to maintain and advance this progress. Keeping pace with population growth, increasing regulations, and upgrading or replacement of existing facilities when their useful design life is reached are ongoing challenges of the SFC program. As noted by IHS in 2015, safe and adequate water supply and/or waste disposal facilities are lacking in approximately 9 percent of AI/AN homes, which is significantly higher when compared to the overall U.S. population [21]. Uneven distribution among tribal communities is also an issue, with the majority of unserved homes in rural locations on large reservations such as the Navajo Nation in Arizona, New Mexico, and Utah, and in remote Alaskan villages.

The creation and ongoing support of the SFC Program is due in large part to a vision of health within IHS that has integrated prevention principles, including public health engineering, rather than promoting a narrower, more clinically focused view of health. As stated in SFC’s annual report, “The IHS considers the provision of sanitation facilities to be a logical extension of its primary health care delivery efforts” [20]. This integration has led to very large scale positive impacts on the health of Native people in the U.S. through prevention programs directed by the DSFC. As stated by Dr. Everett Rhoades, the first Native American Director of IHS (1982–1993), “PL 86–121 had the biggest impact on Indian Health since the smallpox vaccination campaign of the 1830s”. The most evident impacts have been on rates of gastrointestinal disease, which have dropped dramatically in the AI/AN population since the creation of the SFC program [20,22,23]. Recent studies in Alaska have also shown that piped water service was associated with fewer respiratory, skin and soft tissue infections and pneumococcal disease among Alaska Natives [23,24]. In addition, there are other ancillary health benefits from the SFC Program that have not been as well documented. For example, anecdotal evidence from clinical practitioners working on the Navajo Nation indicates that elderly tribal members suffer fewer falls when indoor plumbing is installed and residents are not forced to use outdoor privies, especially during harsh and icy winter conditions.

The success of the SFC Program can be attributed to several factors, including the dedication of significant public resources to the program, including nearly U.S. $1 billion in IHS-provided project funding over the 10 years from fiscal years 2007 to 2016, the commitment and hard work of public health engineers and technicians (even if they would not self-identify as such), consistent tribal input to identify needs and appropriate projects to address them, and long-term institutional vision and commitment to improving sanitation conditions in AI/AN communities over decades.

## 4. Educating Public Health Engineers in the Future

Because engineering and public health have become partially separated as professions and in practice, there is no longer a standardized program of training for public health engineers in the U.S. Few engineers set out initially to work in public health, but that needs to change if we are to effectively address many public health challenges. Engineers working in public health often graduate from “typical” engineering programs, and then gravitate toward public health engineering work either through personal interest or because of unique opportunities. Many engineers come into public health through global work, in which engineering expertise is applied to basic public health problems, such as improving water, sanitation, and hygiene (WASH) conditions in low and middle income countries. Prior work has reviewed the core competencies required for engineers to work in global settings, in addition to making recommendations for educating “globally competent engineers” [2].

In discussing education and curricula for public health engineering, we do not intend to present a comprehensive review of existing programs, but rather some examples of how engineering and public health education can be combined. Neither is our purpose to propose a definitive solution, but rather to pose the question of how best to educate public health engineers. In some fields, such as biomedical and safety engineering, the current form of educating engineers for working in public health fields may be suitable and appropriate. In others, however, a combination of formal education and experience in both engineering and public health are increasingly needed to ensure effective work. We believe this to be the case in the environmental aspects of engineering related to public health, the domain in which we propose to resurrect the term public health engineering.

There are a small number of longstanding graduate programs in the U.S. that combine elements of public health and engineering within schools of public health, including Johns Hopkins University and the University of North Carolina at Chapel Hill. On the other hand, schools of engineering at Tufts University and Columbia University integrate public health into environmental engineering programs. More recently, several programs have more formally integrated elements of public health into schools of engineering, including Stanford University and the Georgia Institute of Technology. This growth has often been at least partially driven by student demand and interest in combining these two fields. That interest is also evident in the growth of Engineers Without Borders (EWB) programs at many U.S. universities. EWB is a voluntary organization of students and professionals that partner with communities to meet basic needs through sustainable engineering projects, a large fraction of which focus on health-related infrastructure.

Although we focus on the U.S. experience in this paper, universities outside the U.S. also have longstanding programs combining engineering and public health. These often focus on water and sanitation issues in lower income countries, and include, for example, programs at the University of Leeds and Loughborough University in the United Kingdom. A more comprehensive review of programs combining public health and engineering, as well as further considerations about potential curricula for public health engineering is beyond the scope of this paper.

In the U.S., we have observed what may be a trend of engineers with an undergraduate engineering degree and a Master of Public Health (MPH) working in the field of public health, although the numbers to date have not been large enough to substantiate any conclusions. According to recent graduates, this combination of academic training is a good one if employers know how to use it to take advantage of and integrate both skill sets.

Whether engineers working in public health obtain their education in both public health and engineering in integrated or separate programs, having such a combined background is optimal to ensuring that they are most effective. A combined background allows them to understand and function across both fields and apply both the engineering and public health skill sets to public health challenges. For example, an engineering education builds critical thinking and problem-solving skills that can be applied to public health issues. Public health education is complementary in that it helps engineers apply those skills in assessing public health impacts, especially in activities involving engineering interventions. Expanding opportunities for students to obtain backgrounds in both fields is also essential for reinvigorating public health engineering as a profession.

## 5. Conclusions: Re-Integrating Public Health Engineering into Practice

The SFC Program within the Indian Health Service discussed above provides an excellent example of a public health engineering program that has positively impacted public health in the U.S. At the same time, since this successful program is not widely known outside of American Indian and Alaska Native communities, it also demonstrates the lack of recognition of public health engineering as a profession. Restoring recognition to public health engineering and expanding its practice will help to lay the foundation for large scale public health improvements.

**Figure 2:**
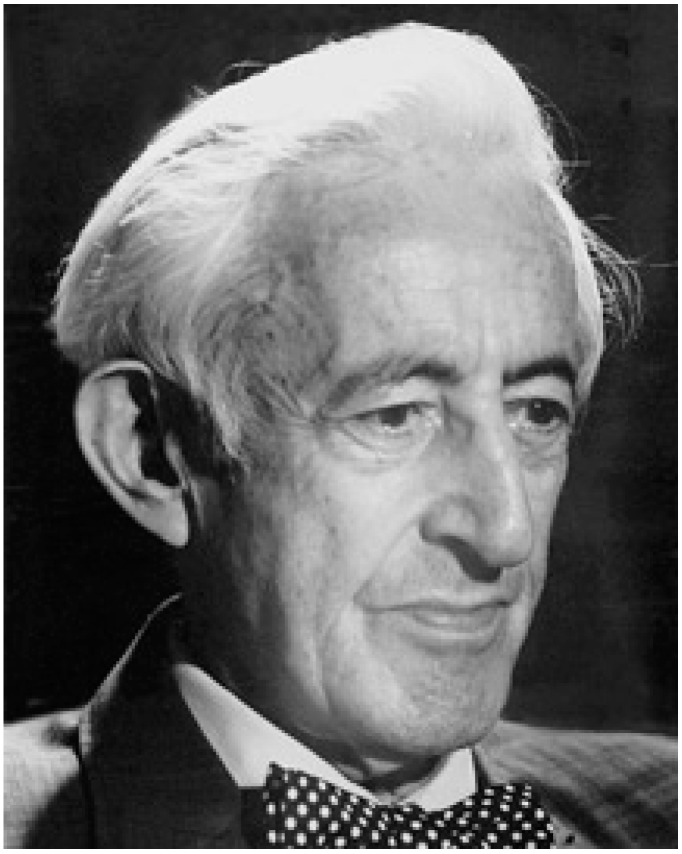
Abel Wolman (Tyler Prize for Environmental Achievement, 2016). (Received consent to publish from Abel Wolman).

In addition, the preceding case highlights many of the attributes of the public health engineer as seen in practice. These qualities were also championed during the extraordinary career of Abel Wolman (Figure 2) and his advocacy for engineering to be integrated in the practice of public health. Wolman was a prolific writer and thought leader in public health engineering, authoring hundreds of papers over the course of a career spanning eight decades [25]. In his last paper, presented to the 1986 World Health Forum when he was 94, Wolman emphasized the importance of the environment as a determinant of health throughout the world [26]. The scale of global health challenges argue for interdisciplinary approaches to public health practice that are very consistent with Wolman’s vision for the application of the engineering skill set to population health challenges around the globe. For example, the case study of the Indian Health Service program discussed in this paper may provide a potential model for improving WASH conditions in other underserved locations, and consequently improving public health. It has been widely recognized that the severity of the cholera epidemic which started in 2010 in Haiti was due to the lack of water and sanitation infrastructure and services in that country [27]. Because the WASH deficiencies in Haiti evolved during decades of limited attention and resources, a long term, sustained public health engineering approach with dedicated resources similar to that of the SFC program will be needed to improve such situations. This would, in turn, require increased development of public health engineers with the necessary skills and experience to manage and sustain such programs. There are some hopeful signs of progress in this area. As mentioned above, university programs incorporating engineering and public health, while still relatively uncommon in the U.S., have expanded in recent years, especially focused on WASH issues in lower income countries. These programs have been met with an overwhelmingly positive response from students, and will help to lay the foundation for a resurgence of engineers working in the public health arena. That will, in turn, underline the need for wider recognition of public health engineering as a profession.

In summary, we believe that there is a current need to strengthen the connection between engineering and the practice of public health, and that an appropriate descriptor for this connection is “public health engineering”.
